# Relationships between Host Phylogeny, Host Type and Bacterial Community Diversity in Cold-Water Coral Reef Sponges

**DOI:** 10.1371/journal.pone.0055505

**Published:** 2013-02-05

**Authors:** Sandra Schöttner, Friederike Hoffmann, Paco Cárdenas, Hans Tore Rapp, Antje Boetius, Alban Ramette

**Affiliations:** 1 HGF-MPG Group for Deep Sea Ecology and Technology, Max Planck Institute for Marine Microbiology, Bremen, Germany; 2 Marine Microbiology Group, Department of Biology, University of Bergen, Bergen, Norway; 3 Centre for Geobiology, Department of Biology, University of Bergen, Bergen, Norway; 4 Department of Biology, University of Bergen, Bergen High-Technology Center, Bergen, Norway; Argonne National Laboratory, United States of America

## Abstract

Cold-water coral reefs are known to locally enhance the diversity of deep-sea fauna as well as of microbes. Sponges are among the most diverse faunal groups in these ecosystems, and many of them host large abundances of microbes in their tissues. In this study, twelve sponge species from three cold-water coral reefs off Norway were investigated for the relationship between sponge phylogenetic classification (species and family level), as well as sponge type (high *versus* low microbial abundance), and the diversity of sponge-associated bacterial communities, taking also geographic location and water depth into account. Community analysis by Automated Ribosomal Intergenic Spacer Analysis (ARISA) showed that as many as 345 (79%) of the 437 different bacterial operational taxonomic units (OTUs) detected in the dataset were shared between sponges and sediments, while only 70 (16%) appeared purely sponge-associated. Furthermore, changes in bacterial community structure were significantly related to sponge species (63% of explained community variation), sponge family (52%) or sponge type (30%), whereas mesoscale geographic distances and water depth showed comparatively small effects (<5% each). In addition, a highly significant, positive relationship between bacterial community dissimilarity and sponge phylogenetic distance was observed within the ancient family of the Geodiidae. Overall, the high diversity of sponges in cold-water coral reefs, combined with the observed sponge-related variation in bacterial community structure, support the idea that sponges represent heterogeneous, yet structured microbial habitats that contribute significantly to enhancing bacterial diversity in deep-sea ecosystems.

## Introduction

Cold-water coral reefs are considered as “biodiversity hotspots” due to their potential to locally enhance faunal biodiversity on continental margins and in the deep ocean [1]–[4]. For this reason, the microbial diversity associated with cold-water reef ecosystems has attracted increasing scientific interest lately [5]–[10]. Marine sponges usually host rich, specifically associated microbial communities that are largely distinct from those of the surrounding seawater [11]–[13]. Representing the most diverse faunal group on cold-water coral reefs [14], these benthic animals are therefore hypothesized to play an important role for promoting microbial diversity in deep-water reef ecosystems.

Recent studies on sponge functional microbiology showed that sponge microbes are able to perform a large suite of aerobic and anaerobic, autotrophic and heterotrophic processes of the nitrogen and sulphur cycle [15]–[17]; the potential main benefits for the sponge thereby being efficient nutrient recirculation and waste product removal. Furthermore, sponges are able to utilize dissolved organic matter (DOM) mediated by their microbes [18]–[19], and have therefore been identified as “DOM-sinks” on tropical coral reefs [20]–[21]. As cold-water corals release considerable amounts of both particulate and dissolved organic matter [22], diverse sponge-microbe associations may also play an important role for the rapid nutrient recirculation in cold-water coral reef systems.

A relation between certain morphological and metabolic features in sponges and the abundance of their associated microbes has been postulated, leading to the distinction of two ecological sponge types [11], [23]–[24]: High-microbial abundance (HMA) sponges, on the one hand, have compact, spherical growth forms, dense tissue with few canals, low pumping rates and a high density of diverse microbial cells. Low-microbial abundance (LMA) sponges, on the other hand, exhibit different growth forms, a loose tissue structure with many canals, high pumping rates and a lower density of microbial cells. Their nutrition is obtained basically by filter feeding, and their tissue contains mainly transient microbial communities.

As indicated by molecular investigations, HMA and LMA sponges differ not only with respect to the density but also the diversity of their microbial communities [23], [25]. Furthermore, several microbial sponge-specific sequence clusters (SSSC) have been identified, which are repeatedly found in different sponge species from different oceans [12], [26]–[27] . Thus, most sponge species, with only few known exceptions, host conspicuously stable, species-specific microbial associates [28]–[34]. These are assumed to interact with their host in many different ways and are commonly referred to as sponge symbionts [35].

Vertical symbiont transmission, one of the microbial acquisition mechanisms by which a temporally and spatially stable association is achieved, has been shown for numerous sponge species belonging to all four classes of Porifera [36]–[40]. Comparative phylogenetic studies of certain host sponges and their symbionts even suggest sponge-microbe co-speciation [41]–[43]. However, horizontal transmission, i.e. the selective and non-selective uptake of microorganisms from the environment, can also occur, including a potential microbial transfer between sponges of the same or different species [40]. A combination of both vertical and horizontal transmission has therefore been proposed to explain the microbial diversity found in sponges today, with increased attention to the environmental component [12]–[13], [34], [44]. As sponges are filter feeders, the additional and constant unspecific uptake of environmental microorganisms can generally obscure the presence and degree of specific sponge-microbe associations.

In cold-water coral reefs, most of the previous studies on sponge-microbe associations either considered only a restricted number of sponge species, or only a certain fraction of the associated microbial community. In the present study, we determined how the diversity of abundant bacterial taxa changed among and between twelve different sponge species, covering a broad phylogenetic spectrum: seven sponge families from three classes of sponges. The high-resolution molecular fingerprinting approach ARISA (Automated Ribosomal Intergenic Spacer Analysis [45]) was chosen due to its better ability to reveal fine community changes, as it targets a genomic region of greater variability than the 16S rRNA gene sequence. By studying samples from three cold-water coral reef ecosystems on the Norwegian continental margin, the following research questions and hypotheses were addressed: (1) Can we identify differences in the number and distribution of bacterial operational taxonomic units (OTUs) depending on sponge phylogeny (family and species) as well as sponge type (HMA *versus* LMA)? (2) How much do the potential host-microbe associations depend on other environmental factors such as geographic location and water depth?

## Results

**Figure 1:**
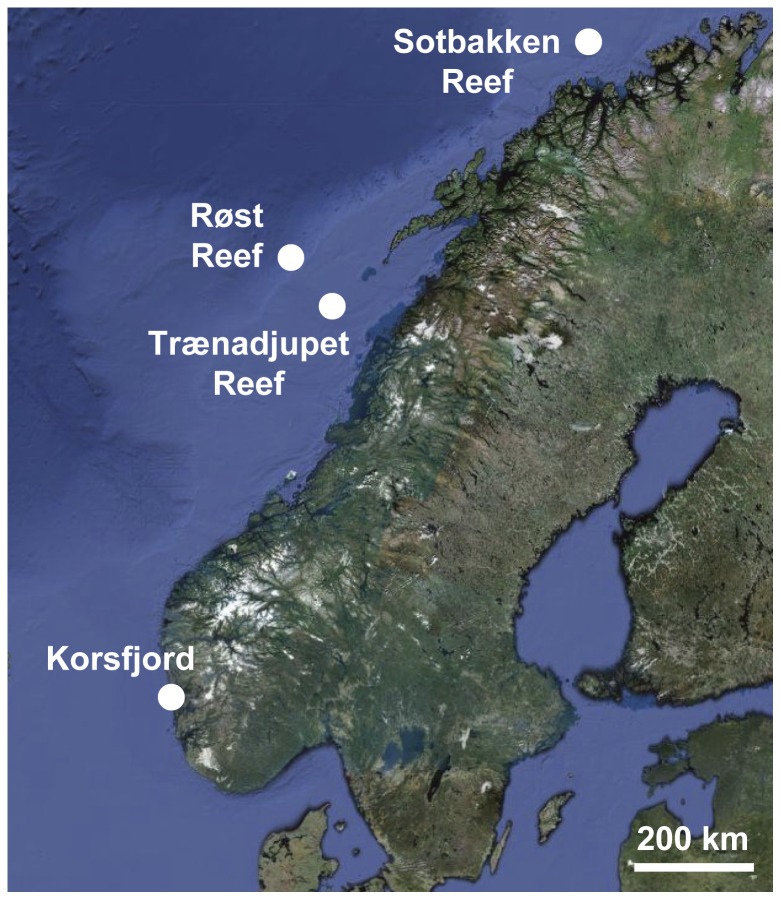
Study sites. Sponges and sediments were sampled at three cold-water coral reef sites off Northern Norway at a water depth of 290–430 m. Additional samples were collected in the Korsfjord on the West coast of Norway at a similar depth range.

**Figure 2:**
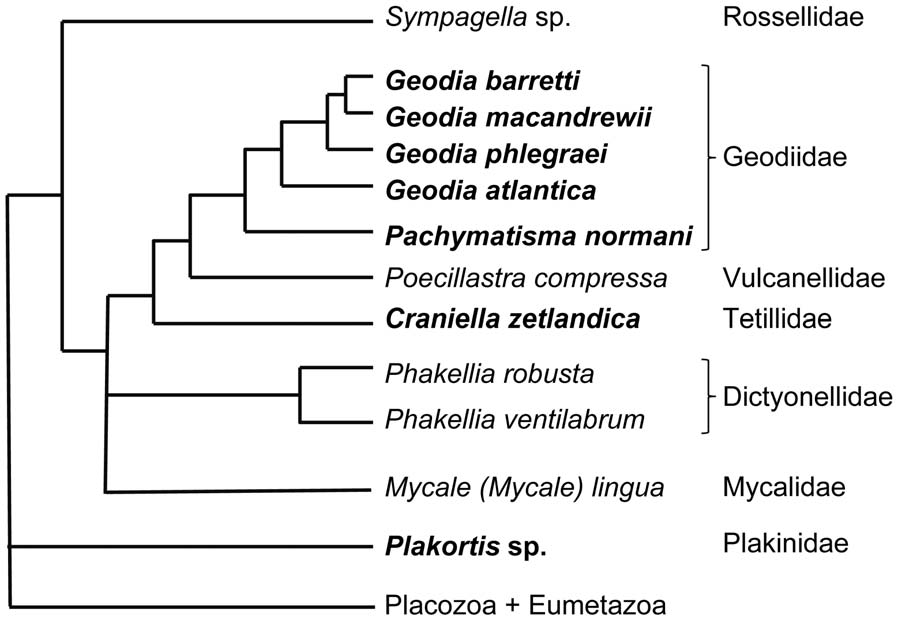
Sponge phylogenetic relationships. The tree topology represents a synthesis of phylogenetic reconstructions from [66], [74]–[75], showing the twelve sponge species from eight different sponge families targeted in this study. Bold: high microbial abundance (HMA) sponges; non-bold: low microbial abundance (LMA) sponges. The concept of LMA and HMA sponges does not follow a phylogenetic trend.

Specimens from 12 sponge species, present at three cold-water coral reef sites off Northern Norway and at a fjord site on the Norwegian West coast (Figure 1), and encompassing a broad range of sponge families and species (Figure 2), were included in this study: The HMA sponges *Geodia barretti*, *Geodia macandrewii*, *Geodia phlegraei*, *Geodia atlantica*, *Pachymatisma normani*, *Plakortis* sp., *and Craniella zetlandica*, as well as the LMA sponges *Poecillastra compressa*, *Phakellia robusta*, *Phakellia ventilabrum*, *Mycale (Mycale) lingua*, and *Sympagella* sp. The different sponge species were grouped into the LMA or HMA sponge type based on microscopic investigation of tissue sections stained with DAPI as described previously [15].

**Figure 3:**
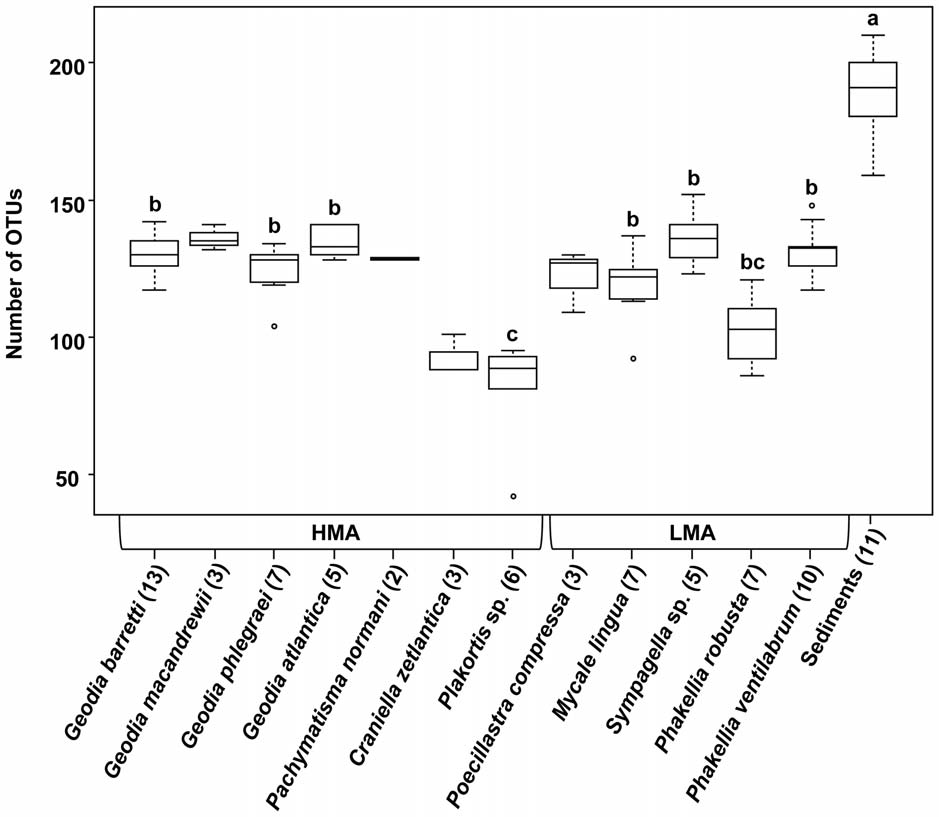
Bacterial OTU numbers. ARISA-derived OTU numbers for all sponge species and ambient sediments are indicated with the number of analyzed samples (i.e. individual sponge specimens) in parentheses. The middle line in each box depicts the median of the respective data set. The box width represents 50% of the data, while both whiskers and outliers indicate the distribution of remaining data points, thus representing the overall variation. Different lower-case letters above boxes denote significant difference in OTU number based on pairwise Kruskal-Wallis rank sum testing at the false detection rate-based adjusted *P*<0.05 [76]. Species with less 5 specimens were not statistically compared.

**Figure 4:**
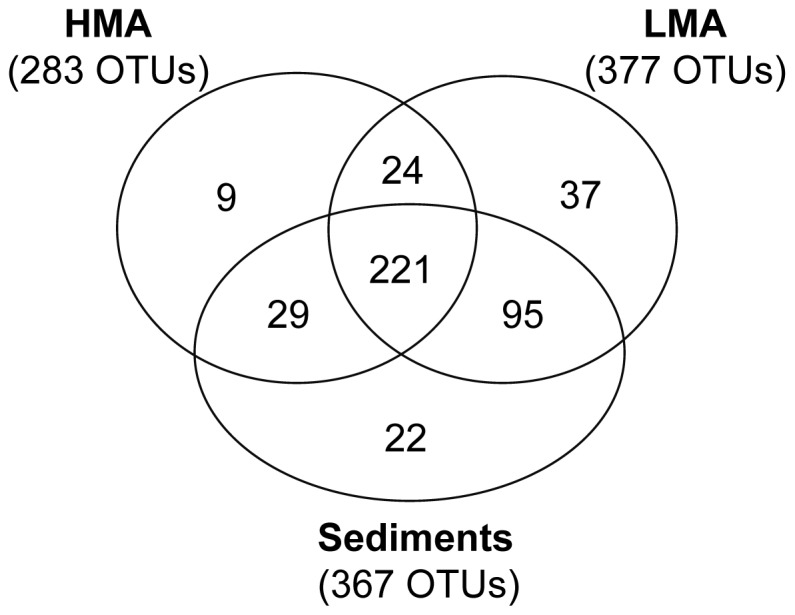
Shared OTUs between HMA sponges, LMA sponges, and sediments. The Venn diagram displays the number of OTUs unique to or shared between the three major sample groupings, with total OTU numbers for each grouping indicated in parentheses.

The whole ARISA dataset yielded a pool of 437 different OTUs, with numbers obtained per sample ranging between 42–210 OTUs. Average OTU numbers (Figure 3) differed significantly between sponges and sediments (range: 42–152 and 159–210, respectively; Kruskal Wallis test, P<0.05), and between some sponge species within and between the HMA and LMA type (range: 42–142 and 86–152 OTUs, respectively; P<0.05). Altogether, HMA sponges (total: 283 OTUs) hosted lower numbers of bacterial OTUs than LMA sponges (total: 377 OTUs) and sediments (total: 367 OTUs; Figure 4). Of all 437 OTUs present in this dataset, 70 OTUs (16%) were strictly associated with sponges and 22 OTUs (5%) were strictly associated with sediments, while a total of 345 OTUs (78.9%) accounted for the shared OTU fraction in sponges and sediments (Figure 4).

**Figure 5:**
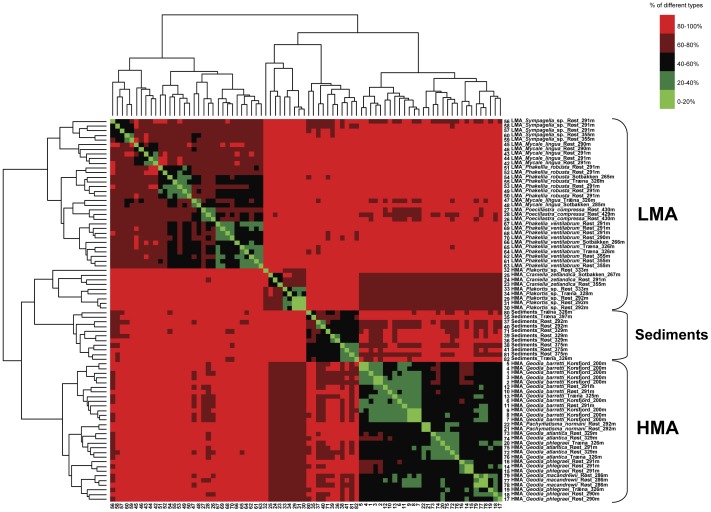
Shared OTUs between all samples. The heatmap depicts the percentage of OTUs shared between any two samples in the study. Community data were also clustered to better reveal the structure in the dataset. Sample names indicate sponge genus, species, reef location, and water depth, as well as specimen identification number.

Only very few of the strictly sponge-specific OTUs were sponge species-specific: The LMA species *P. compressa*, *P. ventilabrum*, and *M. lingua*, harbored 2, 5, and 11 specific OTUs respectively, while the HMA species *C. zetlantica* and *Plakortis* sp. harbored 2 and 22 specific OTUs, respectively. Noticeably, all OTUs found in sediments were also identified at least once in a sponge species. The proportion of shared OTUs between sponges and sediments (on average: 29%) significantly varied among sponge families (ANOVA, df = 6, F = 16.473, P<0.001), as well as sponge species (ANOVA, df = 11, F = 13.980, P<0.001), but not between HMA *versus* LMA sponges (ANOVA, df = 1, F = 0.001, P = 0.977) or reef locations (ANOVA, df = 3, F = 1.8408, P = 0.149) or water depths (ANOVA, df = 1, F = 1.1009, P = 0.298; Figure 5).

**Figure 6:**
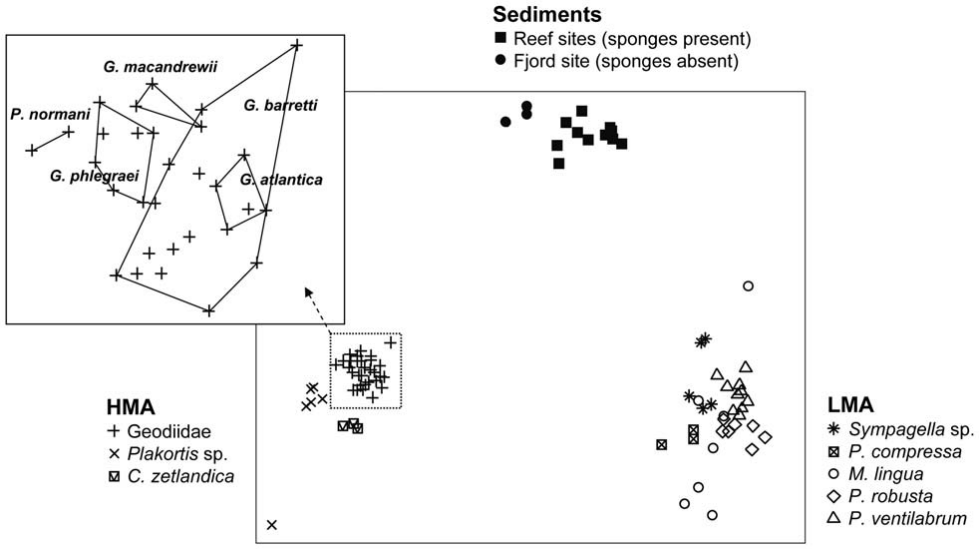
Overall bacterial community variation. The two-dimensional NMDS ordination (based on a Bray-Curtis dissimilarity matrix) of the multidimensional data in reduced space yielded a stress value of 0.11. Each symbol corresponds to one sediment sample or sponge specimen of a given species, with the respective PCR-triplicates already merged into a consensus profile per sample. Insert: Bacterial community variation for specimens from five species of the Geodiidae family.

When variations in bacterial community structure were visualized in a 2-dimensional space using NMDS (Figure 6), communities of HMA and LMA sponges formed clearly distinct groups (ANOSIM R = 0.996, P<0.0001), which also differed greatly from sediment communities (ANOSIM R>0.96 for each comparison, P<0.0001). In addition, within the HMA and LMA groups, bacterial community structures were different between sponge families (ANOSIM R values from 0.557 to 1, all P<0.05 after Bonferroni correction), with the exception of Tetillidae and Pachastrellidae (ANOSIM R = 1, P = 0.10). Furthermore, the five species of the Geodiidae family, although showing rather similar microbial communities when compared to other sponge families (main NMDS plot; Figure 6), also harbored distinct bacterial communities when compared among each other (insert of Figure 6; ANOSIM R = 0.62–1, all P<0.05 after Bonferroni correction, except for *G. barretti versus P. normani* with P = 0.0996). Note that, although the communities from *G. barretti* and *G. atlantica* showed some overlap on the NMDS plot, their bacterial communities were in fact significantly distinct (ANOSIM R = 0.96, P = 0.0001).

**Figure 7:**
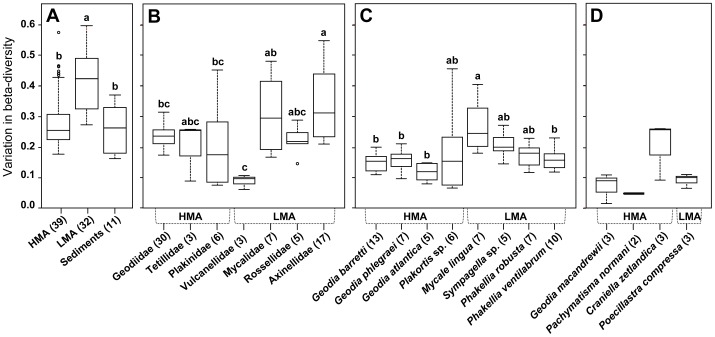
Bacterial community diversification. Variation in beta diversity, measured as the average degree of dispersion (i.e. the average dissimilarity from individual samples to their group centroid) among (**A**) sponge types and ambient sediments, (**B**) sponge families, (**C**) sponge species with more than 5 specimens per species, (**D**) sponge species with less than 5 specimens. Different lower-case letters above each box denote significant mean difference in dispersion based on pairwise Wilcoxon-Mann-Whitney testing at P<0.05. Species with less 5 specimens were not statistically compared.

In order to determine whether the extent of community heterogeneity between samples observed in the NMDS plot was also statistically supported, the degree of community dispersion (i.e. the variability in community diversification or in beta diversity) among sponge types, families, and species were compared by testing whether the average spread within a given NMDS sample grouping was significantly different from that of other sample groupings (Figure 7). As indicated by permutation test for homogeneity of multivariate dispersions (df = 2, F = 19.819, P = 0.001; based on 1000 permutations), significant community diversifications were found between HMA and LMA sponges (Tukey's test, P<0.001), as well as between LMA sponges and sediments (P<0.001), with the LMA sponges showing the overall highest degree of community diversification (Figure 7A). At the level of sponge family (df = 6, F = 5.4838, P = 0.001), subsequent differences in bacterial community dispersion between families were only partly significant (P<0.05), mostly due to the comparatively high degree of bacterial community diversification within the Dictyonellidae and Mycalidae, and the contrastingly low degree of diversification within the Vulcanellidae (Figure 7B). At the level of sponge species (considering those with >5 specimens; df = 11, F = 3.9249, P = 0.001), bacterial community dispersion differed significantly only between the most diversified species, *M. lingua*, and the group of least diversified species, *G. atlantica*, *G. barretti*, *G. phlegraei*, and *P. ventilabrum* (Tukey's test, P<0.05; Figure 7C).

**Figure 8:**
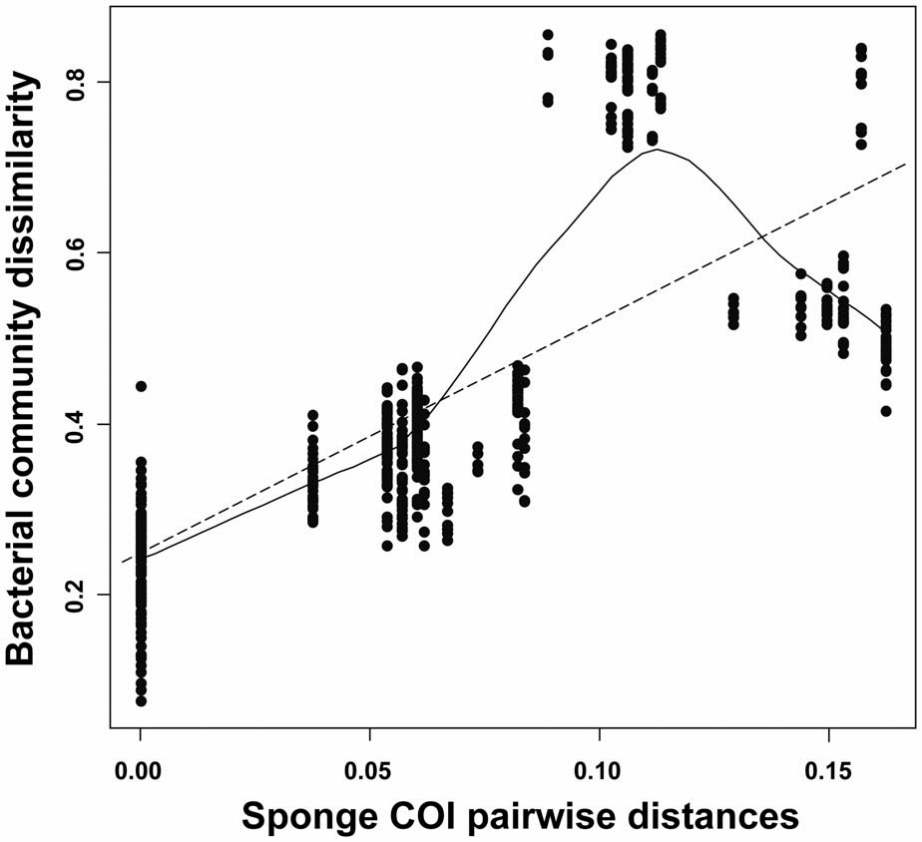
Bacterial community dissimilarity *versus* sponge COI genetic distance within and beyond the Geodiidae family. A highly significant, positive relationship within the Geodiidae (COI genetic distance <0.1) was evidenced (Mantel test), indicating that the more phylogenetically related two Geodiidae species are, the more similar the structure of their bacterial communities, but no such relationship was found at higher sponge taxonomic levels (COI genetic distance >0.1). Both a linear regression line (y = 0.249+2.679 x; dotted line) and a locally weighted polynomial regression curve (LOESS; continuous line) are indicated to better reveal the type of relationship between the data.

When the diversity of bacterial communities in specimens of the Geodiidae family was examined in more detail (Figure 8), a pronounced increase was evidenced at sponge phylogenetic distances, especially up to 0.1 (corresponding to inter-specific variation within the Geodiidae), with a highly significant, positive relationship between bacterial community dissimilarity and sponge phylogenetic distance (Mantel test, R = 0.71 and 0.76, both P<0.001, using OTU relative abundance and presence-absence, respectively).

To systematically examine the relative effects on changes in bacterial community structure of the factors sponge type (HMA *versus* LMA), sponge family and sponge species, as well as the co-factors reef location and water depth, a variation partitioning analysis was performed (Table 1). Sponge type, family and species were all highly significantly associated with changes in bacterial community structure, with sponge species causing most of the variation (63%), followed by sponge family (52%) and sponge type (30%), while the effects of sampling depth and geographic location were controlled for. Interestingly, when this analysis was carried out with only the 245 OTUs shared between HMA and LMA sponges, the respective contribution of each factor did not qualitatively change (Table 1), implying that community variations were mainly based on differences in relative OTU abundance rather than OTU presence/absence. The co-factors water depth (ranging from 200–400 m) and reef location (covering distances of 420 nautical miles within one climatic and geographic region) generally explained less than 5% of the variation in bacterial communities at all levels tested (Table 1).

**Table 1:** Respective effects of sponge type, sponge taxonomy, geographic location and water depth on the structure of sponge-associated bacterial communities.

Effects^a^	df^b^	Explained variance^c^	
		All OTUs	Shared OTUs
Type+Depth+Location	5	0.403 ***	0.237 ***
Type|(Location, Depth)	1	0.303 ***	0.160 ***
Depth|(Location, Type)	1	0.028 ***	0.038 ***
Location|(Type, Depth)	3	0.029 ***	0.048 ***
Family+Depth+Location	10	0.618 ***	0.335 ***
Family|(Location, Depth)	6	0.517 ***	0.259 ***
Depth|(Location, Family)	1	0.012 **	0.006 ns (P = 0.107)
Location|(Family, Depth)	3	0.022 **	0.014 ns (P = 0.0517)
Species+Depth+Location	15	0.733 ***	0.507 ***
Species|(Location, Depth)	11	0.633 ***	0.431 ***
Depth|(Location, Species)	1	0.008 ***	0.001 ns (P = 0.326)
Location|(Species, Depth)	3	0.019 ***	0.024 **

a Simple and partial RDA models were used to quantify the relative effects of sponge type (HMA *versus* LMA), sponge family, and sponge species, in combination with the co-factors reef location and water depth, on changes in bacterial community structure. The “|” sign indicates that the effects of the factors in parenthesis were accounted for by partial regression analysis.

b Degrees of freedom.

c Coefficient of determination (R^2^) of each multivariate model, adjusted for the number of explanatory terms in the respective model. Significances of the *F* ratios, as determined by 1000 Monte Carlo permutation tests, are indicated as ***, P<0.001; **, P<0.01; ns, P≥0.05.

## Discussion

In this study we showed that both HMA and LMA sponges from cold-water coral reefs host diverse bacterial communities in terms of OTU number (used here as proxy for bacterial richness, or alpha diversity), as well as of community structure and diversification (i.e. beta diversity). Community patterns clearly reflected sponge phylogeny/taxonomy and sponge type. High-resolution sequencing applications have become standard tools in microbial ecological studies over the course of the past few years, with the advantage that deep insights into community composition can be obtained. ARISA targets another part of the genome, i.e. the more polymorphic intergenic spacer (ITS1) between the 16S and 23S rRNA genes, and thus offers more resolution than the more conserved ribosomal genes [45]. Therefore our findings cannot be directly compared to 16S-based studies, as finer changes in community structure may not be captured with the latter. Even though molecular fingerprinting techniques do not provide detailed information on the identity and actual number of species in environmental samples, those approaches have been shown to be useful to describe trends and patterns of prokaryotic communities and to respond to a variety of ecological questions at different spatial and temporal scales, across various ecosystems (e.g. [46]–[48]).

### Cold-water coral reef sponges as specific microbial habitats

Altogether 16% of all bacterial OTUs in the dataset appeared sponge-associated (Figure 4), i.e. present only in sponges and not in sediments, with LMA sponges harboring over 3-times more sponge-associated OTUs than HMA sponges. It is tempting to suggest that these OTUs may be sponge-specific, and may even consist of the sponge species-specific microbiome (i.e. “sponge associates” and “sponge specialists”, respectively; *sensu* Taylor [29]), such as revealed by several previous studies [12]–[13], [26], [29], [34], [49]. This finding must, however, be viewed in the light of the fact that only sediments (but not seawater) were included in our study, based on the consideration that sediments function as natural filters of seawater communities in porous sediments [50], and may thus locally accumulate microbes over a longer time span, as compared to what small volumes of seawater may contain at a particular moment (e.g. at sampling time). Furthermore, when we performed our sampling, studies on marine sponge microbes had concordantly suggested sponge-specific microbial communities to be absent from the surrounding seawater (e.g. [11], [12] and references therein). This supposition was refuted only recently, when deep-sequencing efforts revealed the presence of supposedly sponge-specific sequences in the rare seawater biosphere [13], [27], and thereby confirmed hitherto concerns that methodological limitations may have led to inconclusive statements on the acquisition and host-specificity of sponge-associated microbes (see [12] for a thorough discussion on this).

The majority of OTUs (79%) in this study were present in sponges and sediments (Figure 4; i.e. ‘generalists’, *sensu* [20]), with an average of 30% of each sponge's OTU pool shared with sediment-derived OTUs (Figure 5). Although differences in phylogenetic resolution between techniques render direct comparisons difficult, this is in line with a fluorescence in-situ hybridization (FISH)-based study on *Geodia* spp. where a significant overlap between sponge- and sediment-associated microbes was reported [51]. It also reflects findings from deep-sequencing studies that reported the presence of sponge-specific phylotypes in the environment [13], [27]. This shared fraction of the sponge microbiome is assumed to originate from the uptake of seawater-suspended sediment particles by a sponge during filtering, from direct assimilation by amoeboid cells at the sponge surface, or from trapping of sediment particles in surface wounds, which are subsequently overgrown by new tissue ([16] and references therein). In the same way, sponges likely also internalize other seawater-associated bacteria, which then constitute a temporary or permanent part of their microbial community [13], thereby further complicating the identification of a truly sponge-specific community [12].

### Do cold-water coral reef sponges promote local microbial diversity?

Overall, significantly higher bacterial OTU numbers were revealed for sediments compared to sponges (Figure 3), irrespective of sponge species or type. This confirmed the importance of marine sediments as reservoirs of microbial diversity [52]–[53]. The absence of major differences in OTU numbers between HMA and LMA sponges, however, appeared inconsistent with previous assumptions of a higher community complexity in HMA compared to LMA sponges (e.g. [25], [44]). While this could simply be attributed to methodological differences between molecular techniques, it may also indicate that bacterial diversity in LMA sponges from cold-water coral reefs is higher than previously expected, as most of the existing reports are based on sponges from tropical/temperate environments. Furthermore, only 3 sponges (*P. robusta*, *Plakortis* sp., *C. zetlandica*) exhibited lower bacterial OTU numbers than all other species. It must be noted that standard ARISA fingerprinting does not provide a quantitative estimate of the real abundance of bacterial OTUs in nature (see discussions in [54]–[55]), which makes it difficult to extrapolate OTU numbers to absolute bacterial richness, or to population sizes. Nonetheless, the monitoring of relative differences in bacterial OTU number within a given dataset definitely allows meaningful inferences about potential richness dynamics.

In contrast to the weak variability in sponge-associated bacterial OTU numbers, significant variations in community structure were detected between HMA and LMA sponges, and between different sponge species and families (Table 1, Figure 6, Figure 7), suggesting that bacterial community differences in the investigated cold-water sponges manifest mainly at the beta (rather than the alpha) diversity level. This has also been observed for scleractinian cold-water corals from the same reef sites [56], which, compared to these sponges, exhibit a much lower number of bacterial OTUs in their mucus exudates and branch surface biofilms (including coenosarc tissue and calcareous skeleton particles). Interestingly, according to the dispersion results obtained here (Figure 7), bacterial beta diversity in cold-water sponges may be at least as high (HMA sponges) as in the surrounding sediments, if not higher (LMA sponges), which is also the case with cold-water corals (Schöttner, unpublished).

The diversity of sponges on cold-water coral reefs is usually high, with more than 80 species identified on the three reefs covered in this study (Cárdenas, unpublished). Although some of these sponge species may select for partly similar environmental microbes, most of them are assumed to represent distinct microbial habitats. This hypothesis is supported by the recent pyrosequencing study by Schmitt et al. [34], which showed that the core bacterial community, i.e. bacterial OTUs present in the majority of the 32 investigated marine sponge species, is very small (∼1% of total OTUs), while the species-specific community, i.e. bacterial OTUs present in only a single species, is very large (70% of total OTUs). Due to their co-existence within reefs, cold-water sponges may thus, in addition to sediments, increase the availability of different microbial niches and thereby enhance microbial diversity in cold-water coral reef ecosystems.

### Bacterial community structure and sponge phylogeny

Several lines of evidence in this study (Table 1, Figure 5, Figure 6, Figure 7) indicate that host taxonomic classification at the species and family levels are significantly reflected in the structuring of the associated bacterial communities from cold-water coral reefs, with environmental factors such as geographic location or water depth playing only minor roles. In contrast to previous results [34], [40], the importance of sponge phylogeny was clearly reflected for specimens belonging to the Geodiidae family (Figure 8). Up to the Geodiidae family level (COI genetic distance <0.1), a highly significant, positive linear relationship between bacterial community dissimilarity and sponge phylogenetic distance was identified (Figure 8), implying that more closely related Geodiidae species harbor more similar bacterial communities. At higher taxonomic levels (i.e. order or class; COI genetic distance >0.1), however, no such relationship could be evidenced. The absence of trend beyond the family level was further supported by the finding of (i) similar microbial communities being associated with very distantly related sponges, such as the two LMA species *Sympagella* sp. (class: Hexactinellida) and *P. ventilabrum* (class: Demospongiae; Figure 6) and (ii) very different microbial communities being associated with more closely related sponges, such as the LMA species *P. compressa* (order: Astrophorida) and the HMA species of the Geodiidae (order: Astrophorida; Figure 6). This suggests that, beyond the sponge family level, physiological properties depending on e.g. sponge type (HMA *versus* LMA) may be more important for structuring sponge microbial communities than sponge phylogeny. It should be noted though, that sponge phylogeny at higher taxonomic levels is far from being clearly resolved and still subject to ongoing discussions among sponge taxonomists [57].

The positive relationship between sponge phylogeny and bacterial community dissimilarity within the Geodiidae (Figure 8) could be indicative of sponge-bacterial co-evolution within this ancient sponge family for which the fossil record presumably dates back to the Early Cambrian [58]–[59]. Vertical transmission of microbes from adult sponges to larvae has already been evidenced for several sponge species [37]–[40], including members of the Astrophorida genus [60]–[61]. However, the pronounced similarity of bacterial communities within the Geodiidae may just as well reflect a horizontal transmission of microbes, based on the circumstance that all Geodiidae, due to physiological similarities, offer similar microbial niches and thereby may acquire similar microbes from the environment, with the rare seawater (or sediment) biosphere acting as a “seed bank” [13], [35]. Both microbial transmission scenarios seem conceivable here and may even be at play in mixed mode [44], [62], as recently suggested for different other sponge species [13], [34], [40]. Nevertheless, we would like to point out that, if the Geodiidae-associated bacteria in this study were only (or mainly) acquired from the environment, the observed relationship would, very likely, not be so marked, with closely related sponges harboring more similar microbial communities than more distantly related sponges (see also comment in [34]). It remains to be seen whether this particular relationship, here based on *Geodia* spp. specimens from cold-water coral reef ecosystems off Norway, also holds true for other sponge families and respective sponge samples from shallow-water and/or tropical sites.

### Conclusion

This study is the first that considers twelve species of cold-water coral reef sponges to investigate the relationship between sponge phylogeny, sponge type (HMA *versus* LMA), and bacterial community diversity, while taking into account potential effects from geographic location and water depth. Our findings, based on the high-resolution molecular fingerprinting approach ARISA, revealed that a high proportion of the detected bacterial OTUs were shared between different sponges and ambient sediments, while only a lower proportion appeared sponge-associated. Bacterial community structure clearly reflected both sponge phylogeny and sponge type, whereas mesoscale geographic distances and water depth played only a minor role in determining sponge-associated bacterial diversity. Furthermore, a highly significant relationship was observed between bacterial community dissimilarity and sponge phylogenetic distance within the ancient sponge family of the Geodiidae, which raised the question whether partly vertical symbiont transmission could be at play, or only horizontal acquisition from the environment. Future research that combines high-resolution molecular techniques, such as deep-sequencing, with cultural and biogeochemical approaches, will certainly help identify the most likely scenario of the two, if not even their concerted or cumulative effects, and further elucidate the role of sponge-microbe associations in cold-water coral reef ecosystems.

## Methods

### Sampling

Sponges were sampled in June 2007 during the RV Polarstern expedition ARKXXII/1a with the manned submersible JAGO (IfM Geomar) (Røst reef: 67°30,54′N; 9°25,00′E, 290–360 m; Trænadjupet reef: 66°58.21′N; 11°7.56′E, 290–330 m; Sotbakken reef: 70°45,43′N; 18°40,60′E, 260–300 m; Figure 1). Each specimen was collected with the submersible's manipulator arm to avoid damage and contamination with the surrounding sediments. Further sponges were sampled in October 2006, from a hard bottom slope devoid of sediments in the Korsfjord close to Bergen (60°09.20′N; 05°08.86′E, 200–400 m), using a triangular dredge from aboard the RV Hans Brattstrøm. Depending on sample availability, 3–7 specimens per sponge species were collected. Right after retrieval, subsamples of inner sponge tissue were taken with a sterile scalpel, fixed and washed once in 99% ethanol, and frozen at −20°C until further processing. Surface sediments were collected at each sponge sampling location on the three reefs (Røst, Trænadjupet, Sotbakken) as well as at a control site devoid of sponges in the Korsfjord, and immediately frozen at −20°C until further processing.

### DNA extraction and ARISA

Prior to DNA extraction, sponge tissue from each individual specimen was ground with mortar and pestle, and left at room temperature for several minutes to evaporate the ethanol. Total community DNA was extracted from 1 g of either ground sponge tissue or sediments, and purified using the Fast DNA Spin Kit for Soil (BIO 101) according to the manufacturer's instructions. Universal bacterial ARISA using normalized DNA quantities of 50–100 ng per reaction for all samples was performed in triplicates using the primers ITSF and the HEX-labeled ITSReub [63]. ARISA PCR, fragment analysis and processing of ARISA profiles were carried out as described elsewhere [50], [55].

### Sponge identification and sequencing

All sponge specimens (see Supporting Information, Text S1), were identified by PC and HTR, from which permanent spicule preparations are available upon request. The specimens of *P. compressa* and *P. normani* collected during this study have been previously described [64]–[65]. According to a revision of the Astrophorida using molecular data, *P. compressa* is now part of the Vulcanellidae family [66]. *Geodia* and *C. zetlandica* specimens collected during this study are currently used for revisions of *Geodia* and Tetillidae, to be published in forthcoming papers. *P. ventilabrum*, *P. robusta* and *M. (M.) lingua* were identified based on the external morphology and spicule morphology. Since the Axinellidae have been shown to be a polyphyletic family, *P. ventilabrum* and *P. robusta* are for now considered part of the Dictyonellidae [67]. *Plakortis* sp. and *Sympagella* sp. were identified using the identification keys of the *Systema Porifera* [68]. The identification of *Plakortis* sp. was further confirmed by A. Ereskovsky (personal communication), albeit not down to the species level, since this genus is in dire need of revision.

Sequences of the Folmer fragment of the mitochondrial cytochrome *c* oxidase subunit I (COI) gene of some specimens were obtained in previous phylogenetic studies and released under the following GenBank accession numbers: *Geodia* spp. (EU442194, EU442195, EU442198, EU442196), *P. normani* (EF564322), *P. compressa* (EU442192) [64], [66], [69]. Further, using the protocol described in Cárdenas et al. [69], the COI Folmer fragment of *C. zetlandica* (KC122679) was obtained, but could not be retrieved for any of the other species (*P. ventilabrum*, *P. robusta*, *M. (M.) lingua*, *Sympagella* sp., *Plakortis* sp.). Hence, only the Tetractinellida sponges, i.e. 7 out of 12 species targeted in this study, were used for correlating sponge COI genetic distance with changes in bacterial community structure: the 5 Geodiidae species *G. barretti*, *G. atlantica*, *G. macandrewii*, *G. phlegraei*, *P. normani*, as well as the Tetillidae species *C. zetlandica* and the Vulcanellidae species *P. compressa*.

### Multivariate statistics

Total numbers of bacterial operational taxonomic units (OTUs, i.e. binned ARISA peaks) per sponge species were compared for mean difference by applying pairwise Kruskal-Wallis rank sum tests (Figure 3). Numbers and/or percentages of shared OTUs between *a posteriori* sample groupings or single samples were represented by Venn (Figure 4) and heatmap diagrams (Figure 5; including complete linkage cluster analysis), respectively, and tested for significance by performing Monte Carlo permutation tests with 1000 permutations. Community dissimilarity matrices based on the Bray-Curtis index were visualized by Non-metric MultiDimensional Scaling (NMDS; Figure 6), and *a posteriori* sample groupings were tested for significant differences by Analysis of Similarity (ANOSIM) tests (reviewed in e.g. [70]). Since the data consisted of different numbers of sponge specimens per species and reef location, larger groups were re-sampled with the size of the smaller groups to assess the effects of varying data set sizes. In addition, *a posteriori* sample groupings were examined for differences in beta diversity (commonly defined as the variability in community composition between samples) by comparing their average degree of dispersion, i.e. the average dissimilarity from individual samples to the group centroid (visible as spread within NMDS sample groupings), and tested for significance by applying a Monte Carlo permutation test of the data, followed by pairwise Tukey tests (Figure 7).

Furthermore, variation partitioning based on simple and partial redundancy analyses (RDA; Table 1) was performed in order to assess the significance of the respective effects of sponge type and sponge taxonomic classification, in combination with reef location and water depth, on changes in bacterial community structure. Significance tests were based on 1000 Monte Carlo permutations of the simple or partial multivariate models. All response data were Hellinger-transformed prior to analyses [70]–[71]. The relationship between ARISA-derived bacterial community dissimilarity and sponge COI genetic distance (Jukes-Cantor distance matrix calculated in MEGA v.4.0 [72]) was visualized by locally weighted polynomial regression (LOESS) curves (Figure 8), with the strength and significance of the identified trend being evaluated by a Mantel test (based on Pearson's product-moment correlation) with 1000 Monte Carlo matrix permutations. All described analyses were implemented with the statistical platform R v.2.14 using the ‘vegan’ package [73] and custom R scripts.

## Supporting Information

Text S1 **Sponge species (along with their field number) used in this study.** **A.** *Geodia barretti*, PS70/40-4(1). **B.** *Geodia atlantica*, PS70/27-1(12). **C.** *Geodia phlegraei*, PS70/27-1(6). **D.** *Geodia macandrewii*, PS70/15(1). **E.** *Pachymatisma normani*, PS70/13-1(1). **F.** *Craniella zetlandica*, PS70/9-4(6). **G.** *Poecillastra compressa*, PS70/19-7(1). **H.** *Plakortis* sp., PS70/13-1(4). **I.** *Mycale (Mycale) lingua*, PS70/14-4(8). **J.** *Sympagella* sp., PS70/14-4(12). **K.** *Phakellia robusta*, PS70/27-1(1), *Phakellia ventilabrum*, PS70/27-1(2) and PS70/27-1(3). **L.** *Phakellia robusta*, PS70/27-1(1).
(DOC)

## References

[1] Jensen A, Frederiksen R (1992) The fauna associated with the bank-forming deepwater coral *Lophelia pertusa* (Scleractinaria) on the Faroe Shelf. Sarsia 77: 53–69.

[2] Freiwald A, Fosså JG, A, Koslow T, Roberts J (2004) Cold-water coral reefs. Cambridge: UNEP-WCMC. 86 p.

[3] Roberts JM, Wheeler AJ, Freiwald A (2006) Reefs of the deep: The biology and geology of cold-water coral ecosystems. Science 312: 543–547. 16645087 10.1126/science.1119861

[4] Buhl-Mortensen L, Vanreusel A, Gooday AJ, Levin LA, Priede IG, et al (2010) Biological structures as a source of habitat heterogeneity and biodiversity on the deep ocean margins. Mar Ecol 31: 21–50.

[5] Yakimov MM, Cappello S, Crisafi E, Tursi A, Savini A, et al (2006) Phylogenetic survey of metabolically active microbial communities associated with the deep-sea coral *Lophelia pertusa* from the Apulian plateau, Central Mediterranean Sea. Deep Sea Res Part I Oceanogr Res Pap 53: 62–75.

[6] Jensen S, Neufeld JD, Birkeland NK, Hovland M, Murrell JC (2008) Insights into the microbial community structure of a Norwegian deep-water coral reef environment. Deep-Sea Res I 55: 1554–1563.

[7] Neulinger S, Järnegren J, Ludvigsen M, Lochte K, Dullo W (2008) Phenotype-specific bacterial communities in the cold-water coral *Lophelia pertusa* (Scleractinia) and their implications for the coral's nutrition, health and distribution. Appl Environ Microbiol 74: 7272–7285. 18849454 10.1128/AEM.01777-08 PMC2592914

[8] Hanson L, Agis M, Maier C, Weinbauer M (2009) Community composition of bacteria associated with cold-water coral *Madrepora oculata*: within and between colony variability. Mar Ecol Prog Ser 397: 89–102.

[9] Kellogg C, Lisle J, Galkiewicz J (2009) Culture-independent characterization of bacterial communities associated with the cold-water coral *Lophelia pertusa* in the Northeastern Gulf of Mexico. Appl Environ Microbiol 75: 2294–2303. 19233949 10.1128/AEM.02357-08 PMC2675238

[10] Schöttner S, Hoffmann F, Wild C, Rapp HT, Boetius A, et al (2009) Inter- and intra-habitat bacterial diversity associated with cold-water corals. ISME J 3: 756–759. 19279671 10.1038/ismej.2009.15

[11] Hentschel U, Fieseler L, Wehrl M, Gernert C, Steinert M, et al.. (2003) Microbial diversity of marine sponges. In: Müller WEG, editor. Marine Molecular Biotechnology. Berlin: Springer. pp. 59–88. 10.1007/978-3-642-55519-0_3 15825640

[12] Taylor MW, Radax R, Steger D, Wagner M (2007) Sponge-associated microorganisms: evolution, ecology, and biotechnological potential. Microbiol Mol Biol Rev 71: 259–347. 10.1128/MMBR.00040-06 PMC1899876 17554047

[13] Webster N, Taylor M, Behnam F, Lucker S, Rattei T, et al (2010) Deep sequencing reveals exceptional diversity and modes of transmission of bacterial sponge symbionts. Environ Microbiol 12: 2070–2082. 21966903 10.1111/j.1462-2920.2009.02065.x PMC2936111

[14] Soest RWMv, Lavaleye MSS (2005) Diversity and abundance of sponges in bathyal coral reefs of Rockall Bank, NE Atlantic, from boxcore samples. Mar Biol Res 1: 338–349.

[15] Schläppy M-L, Schöttner SI, Lavik G, Kuypers M, de Beer D, et al (2010) Evidence of nitrification and denitrification in high and low microbial abundance sponges. Mar Biol 157: 593–602. 24391241 10.1007/s00227-009-1344-5 PMC3873014

[16] Hoffmann F, Larsen O, Thiel V, Rapp HT, Pape T, et al (2005) An anaerobic world in sponges. Geomicrobiol J 22: 1–10.

[17] Hoffmann F, Radax R, Woebken D, Holtappels M, Lavik G, et al (2009) Complex nitrogen cycling in the sponge *Geodia barretti* . Environ Microbiol 11: 2228–2243. 19453700 10.1111/j.1462-2920.2009.01944.x

[18] Yahel G, Sharp JH, Marie D, Hase C, Genin A (2003) In situ feeding and element removal in the symbiont-bearing sponge *Theonella swinhoei*: Bulk DOC is the major source for carbon. Limnol Oceanogr 48: 141–149.

[19] de Goeij JM, Moodley L, Houtekamer M, Carballeira NM, van Duyl FC (2008a) Tracing 13C-enriched dissolved and particulate organic carbon in *Halisarca caerulea*, a coral reef sponge with associated bacteria: evidence for DOM-feeding. Limnol Oceanogr 53: 1376–1386.

[20] de Goeij JM, van Duyl FC (2007) Coral cavities are sinks of dissolved organic carbon (DOC). Limnol Oceanogr 52: 2608–2617.

[21] de Goeij JM, van den Berg H, van Oostveen MM, Epping EHG, van Duyl FC (2008b) Major bulk dissolved organic carbon (DOC) removal by encrusting coral reef cavity sponges. Mar Ecol Prog Ser 357: 139–151.

[22] Wild C, Mayr C, Schöttner S, Wehrmann L, Naumann M, et al (2008) Organic matter release by cold water corals and its implication for fauna-microbe interaction. Mar Ecol Prog Ser 372: 67–75.

[23] Weisz JB, Hentschel U, Lindquist N, Martens CS (2007) Linking abundance and diversity of sponge-associated microbial communities to metabolic differences in host sponges. Mar Biol 152: 475–483.

[24] Weisz JB, Lindquist N, Martens CS (2008) Do associated microbial abundances impact marine demosponge pumping rates and tissue densities? Oecologia 155: 367–376. 18030495 10.1007/s00442-007-0910-0

[25] Kamke J, Taylor MW, Schmitt S (2010) Activity profiles for marine sponge-associated bacteria obtained by 16S rRNA vs. 16S rRNA gene comparisons. ISME J 4: 498–508. 20054355 10.1038/ismej.2009.143

[26] Hentschel U, Hopke J, Horn M, Friedrich AB, Wagner M, et al (2002) Molecular evidence for a uniform microbial community in sponges from different oceans. Appl Environ Microbiol 68: 4431–4440. 12200297 10.1128/AEM.68.9.4431-4440.2002 PMC124103

[27] Simister RL, Deines P, Botté ES, Webster NS, Taylor MW (2012) Sponge-specific clusters revisited: a comprehensive phylogeny of sponge-associated microorganisms. Environ Micobiol 14: 517–524. 10.1111/j.1462-2920.2011.02664.x 22151434

[28] Thoms C, Horn M, Wagner M, Hentschel U, Proksch P (2003) Monitoring microbial diversity and natural product profiles of the sponge *Aplysina cavernicola* following transplantation. Mar Biol 142: 685–692.

[29] Taylor MW, Schupp PJ, Dahllof I, Kjelleberg S, Steinberg PD (2004) Host specificity in marine sponge-associated bacteria, and potential implications for marine microbial diversity. Environ Microbiol 6: 121–130. 14756877 10.1046/j.1462-2920.2003.00545.x

[30] Hill M, Hill A, Lopez N, Harriot O (2006) Sponge-specific bacterial symbionts in the Caribbean sponge, *Chondrilla nucula* (Demospongiae, Chondrosida). Mar Biol 148: 1221–1230.

[31] Wichels A, Wurtz S, Dopke H, Schutt C, Gerdts G (2006) Bacterial diversity in the breadcrumb sponge *Halichondria panicea* (Pallas). FEMS Microbiol Ecol 56: 102–118. 16542409 10.1111/j.1574-6941.2006.00067.x

[32] Thiel V, Leininger S, Schmaljohann R, Brummer F, Imhoff JF (2007) Sponge-specific bacterial associations of the Mediterranean sponge *Chondrilla nucula* (Demospongiae, Tetractinomorpha). Microb Ecol 54: 101–111. 17364249 10.1007/s00248-006-9177-y

[33] Lee OO, Wong YH, Qian P-Y (2009) Inter- and intra-specific variations of bacterial communities associated with marine sponges from San Juan Island, Washington, USS. Appl Environ Microbiol 75: 3513–3521. 19363076 10.1128/AEM.00002-09 PMC2687283

[34] Schmitt S, Tsai P, Bell J, Fromont J, Ilan M, et al (2012) Assessing the complex sponge microbiota: core, variable and species-specific bacterial communities in marine sponges. ISME J 6: 654–576. 10.1038/ismej.2011.116 PMC3280146 21993395

[35] Webster NS, Taylor MW (2012) Marine sponges and their microbial symbionts: love and other relationships. Environ Microbiol 14: 335–346. 21443739 10.1111/j.1462-2920.2011.02460.x

[36] Ereskovsky AV, Gonobobleva E, Vishnyakov A (2005) Morphological evidence for vertical transmission of symbiotic bacteria in the viviparous sponge *Halisarca dujardini* Johnston (Porifera, Demospongiae, Halisarcida). Mar Biol 146: 869–875.

[37] Enticknap JJ, Kelly M, Peraud O, Hill RT (2006) Characterization of a culturable alphaproteobacterial symbiont common to many marine sponges and evidence for vertical transmission via sponge larvae. Appl Environ Microbiol 72: 3724–3732. 16672523 10.1128/AEM.72.5.3724-3732.2006 PMC1472332

[38] Schmitt S, Weisz JB, Lindquist N, Hentschel U (2007) Vertical transmission of a phylogenetically complex microbial consortium in the viviparous sponge *Ircinia felix* . Appl Environ Microbiol 73: 2067–2078. 17277226 10.1128/AEM.01944-06 PMC1855684

[39] Sharp K, Eam B, Faulkner D, Haygood M (2007) Vertical Transmission of Diverse Microbes in the Tropical Sponge *Corticium* sp. Appl Environ Microbiol 73: 622–629. 17122394 10.1128/AEM.01493-06 PMC1796987

[40] Schmitt S, Angermeier H, Schiller R, Lindquist N, Hentschel U (2008) Molecular microbial diversity survey of sponge reproductive stages and mechanistic insights into vertical transmission of microbial symbionts. Applied and Environmental Microbiology 74: 7694–7708. 18820053 10.1128/AEM.00878-08 PMC2607154

[41] Erpenbeck D, Breeuwer AJ, van der Velde HC, Van Soest RWM (2002) Unravelling host and symbiont phylogenies of halichondrid sponges (Demospongiae, Porifera) using a mitochondrial marker. Mar Biol 141: 377–386.

[42] Thacker RW, Starnes S (2003) Host specificity of the symbiotic cyanobacterium *Oscillatoria spongelia* in marine sponges, *Dysidea* spp. Mar Biol 142: 643–648.

[43] Holmes B, Blanch H (2007) Genus-specific associations of marine sponges with group I crenarchaeota. Mar Biol 150: 759–772.

[44] Hentschel U, Piel J, Degnan SM, Taylor MW (2012) Genomic insights into the marine sponge microbiome. Nat Rev Microbiol 10: 641–654. 22842661 10.1038/nrmicro2839

[45] Fisher MM, Triplett EW (1999) Automated approach for ribosomal intergenic spacer analysis of microbial diversity and its application to freshwater bacterial communities. Appl Environ Microbiol 65: 4630–4636. 10508099 10.1128/aem.65.10.4630-4636.1999 PMC91617

[46] Bienhold C, Boetius A, Ramette A (2012) The energy-diversity relationship of complex bacterial communities in Arctic deep-sea sediments. ISMEJ 6: 724–732. 10.1038/ismej.2011.140 PMC3309351 22071347

[47] Gobet A, Böer SI, Huse SM, van Beusekom JEE, Quince C, et al (2012) Diversity and dynamics of rare and of resident bacterial populations in coastal sands. ISMEJ 6: 542–553. 10.1038/ismej.2011.132 PMC3280144 21975598

[48] Fuhrman JA, Hewson I, Schwalbach MS, Steele JA, Brown MV, et al (2006) Annually reoccurring bacterial communities are predictable from ocean conditions. Proc Nat Acad Sci USA 103: 13104–13109. 16938845 10.1073/pnas.0602399103 PMC1559760

[49] Lee OO, Wang Y, Yang J, Lafi FF, Al-Suwailem A, et al (2011) Pyrosequencing reveals highly diverse and species-specific microbial communities in sponges from the Red Sea. ISME J 5: 650–664. 21085196 10.1038/ismej.2010.165 PMC3105750

[50] Böer S, Hedtkamp S, van Beusekom JEE, Fuhrman JA, Boetius A, et al (2009) Time- and sediment depth-related variations of bacterial diversity and community structure in subtidal sands. ISME J 3: 780–791. 19340087 10.1038/ismej.2009.29

[51] Brück WM, Brück TB, Self WT, Reed JK, Nitecki SS, et al (2010) Comparison of the anaerobic microbiota of deep-water *Geodia* spp. and sandy sediments in the Straits of Florida. ISME J 4: 686–699. 20090787 10.1038/ismej.2009.149

[52] Schöttner S, Pfitzner B, Grünke S, Rasheed M, Wild C, et al (2011) Drivers of bacterial diversity dynamics in permeable carbonate and silicate coral reef sands from the Red Sea. Environmental Microbiology 13: 1815–1826. 21554515 10.1111/j.1462-2920.2011.02494.x PMC3207121

[53] Zinger L, Amaral-Zettler LA, Fuhrman JA, Horner-Devine MC, Huse SM, et al (2011) Global patterns of bacterial beta-diversity in seafloor and seawater ecosystems. PLoS ONE 6: e24570. 21931760 10.1371/journal.pone.0024570 PMC3169623

[54] Bent SJ, Forney LJ (2008) The tragedy of the uncommon: understanding limitations in the analysis of microbial diversity. The ISME Journal 2: 689–695. 18463690 10.1038/ismej.2008.44

[55] Ramette A (2009) Quantitative community fingerprinting methods for estimating the abundance of operational taxonomic units in natural microbial communities. Appl Environ Microbiol 75: 2495–2505. 19201961 10.1128/AEM.02409-08 PMC2675222

[56] Schöttner S, Wild C, Hoffmann F, Boetius A, Ramette A (2012) Spatial scales of bacterial diversity in cold-water coral reef ecosystems. PLoS ONE 7: e32093. 22403625 10.1371/journal.pone.0032093 PMC3293894

[57] Cárdenas P, Pérez T, Boury-Esnault N (2012) Sponge systematics facing new challenges. Adv Mar Biol 61: 29–209. 10.1016/B978-0-12-387787-1.00010-6 22560778

[58] Reitner J, Mehl D (1995) Early paleozoic diversification of sponges: new data and evidences. Geol Paläontol Mitt Innsbruck 20: 335–347.

[59] Pisera A (2006) Paleontology of sponges – a review. Can J Zool 84: 242–261.

[60] Sciscioli M, Scalera Liaci L, Lepore E, Gherardi M, Simpson TL (1991) Ultrastructural study of the mature egg of the marine sponge *Stelletta grubii* (Porifera Demospongiae). Mol Reprod Dev 28: 346–350. 2064777 10.1002/mrd.1080280406

[61] Sciscioli M, Lepore E, Gherardi M, Scalera Liaci L (1994) Transfer of symbiotic bacteria in the mature oocyte of *Geodia cydonium* (Porifera, Demospongiae): an ultrastructural study. Cah Biol Mar 35: 471–478.

[62] Bright M, Bulgheresi S (2010) A complex journey: transmission of microbial symbionts. Nat Rev Microbiol 8: 218–230. 20157340 10.1038/nrmicro2262 PMC2967712

[63] Cardinale M, Brusetti L, Quatrini P, Borin S, Puglia AM, et al (2004) Comparison of different primer sets for use in automated ribosomal intergenic spacer analysis of complex bacterial communities. Appl Environ Microbiol 70: 6147–6156. 15466561 10.1128/AEM.70.10.6147-6156.2004 PMC522057

[64] Cárdenas P, Xavier J, Tendal OS, Schander C, Rapp HT (2007) Redescription and resurrection of *Pachymatisma normani* (Demospongiae, Geodiidae), with remarks on the genus *Pachymatisma* . J Mar Biol Assoc UK 87: 1511–1525.

[65] Cárdenas P, Rapp HT (2012) A review of Norwegian streptaster-bearing Astrophorida (Porifera: *Demospongiae: Tetractinellida*), new records and a new species. Zootaxa 3253: 1–53.

[66] Cárdenas P, Xavier JR, Reveillaud J, Schander C, Rapp HT (2011) Molecular phylogeny of the Astrophorida (Porifera, *Demospongiae*) reveals an unexpected high level of spicule homoplasy. PLoS ONE 6: e18318. 21494664 10.1371/journal.pone.0018318 PMC3072971

[67] Morrow CC, Picton BE, Erpenbeck D, Boury-Esnault N, Maggs CA, et al (2012) Congruence between nuclear and mitochondrial genes in Demospongiae: A new hypothesis for relationships within the G4 clade (Porifera: Demospongiae). Mol Phylogenet Evol 62: 174–190. 22001855 10.1016/j.ympev.2011.09.016

[68] Hooper JNA, van Soest RWM (2002) Systema Porifera. A guide to the classification of sponges. New York: Kluwer Academic/Plenum Publishers. 1708 pp.

[69] Cárdenas P, Rapp HT, Schander C, Tendal OS (2010) Molecular taxonomy and phylogeny of the Geodiidae (Porifera, *Demospongiae*, Astrophorida) - combining phylogenetic and Linnaean classification. Zool Scr 39: 89–106.

[70] Ramette A (2007) Multivariate analyses in microbial ecology. FEMS Microbiol Ecol 62: 142–160. 17892477 10.1111/j.1574-6941.2007.00375.x PMC2121141

[71] Legendre P, Gallagher ED (2001) Ecologically meaningful transformations for ordination of species data. Oecologia 129: 271–280. 28547606 10.1007/s004420100716

[72] Tamura K, Dudley J, Nei M, Kumar S (2007) MEGA4: Molecular Evolutionary Genetics Analysis (MEGA) software version 4.0. Molecular Biology and Evolution 24: 1596–1599. 17488738 10.1093/molbev/msm092

[73] Oksanen J, Blanchet FG, Kindt R, Legendre P, O'Hara R B, et al.. (2010) vegan: Community Ecology Package. R package version 1.17-4.

[74] Nichols SA (2005) An evaluation of support for order-level monophyly and interrelationships within the class Demospongiae using partial data from the large subunit rDNA and cytochrome oxidase subunit I. Mol Phylogenet Evol 34: 81–96. 15579383 10.1016/j.ympev.2004.08.019

[75] Sperling EA, Peterson KJ, Pisani D (2009) Phylogenetic-signal dissection of nuclear housekeeping genes supports the paraphyly of sponges and the monophyly of Eumetazoa. Mol Biol Evol 26: 2261–2274. 19597161 10.1093/molbev/msp148

[76] Benjamin Y, Hochberg Y (1995) Controlling the false discovery rate—a practical and powerful approach to multiple testing. J R Stat Soc Ser B 57: 289–300.

