# Supplementary material for: Relationships between Host Phylogeny, Host Type and Bacterial Community Diversity in Cold-Water Coral Reef Sponges
Source: PLoS One. 2013 Feb 5;8(2):e55505. doi: 10.1371/journal.pone.0055505 (PMC3564759; doi:10.1371/journal.pone.0055505)
Supplement: Text S1 — Sponge species (along with their field number) used in this study. A. Geodia barretti, PS70/40-4(1). B. Geodia atlantica, PS70/27-1(12). C. Geodia phlegraei, PS70/27-1(6). D. Geodia macandrewii, PS70/15(1). E. Pachymatisma normani, PS70/13-1(1). F. Craniella zetlandica, PS70/9-4(6). G. Poecillastra compressa, PS70/19-7(1). H. Plakortis sp., PS70/13-1(4). I. Mycale (Mycale) lingua, PS70/14-4(8). J. Sympagella sp., PS70/14-4(12). K. Phakellia robusta, PS70/27-1(1), Phakellia ventilabrum, PS70/27-1(2) and PS70/27-1(3). L. Phakellia robusta, PS70/27-1(1). (DOC) [file pone.0055505.s001.doc]

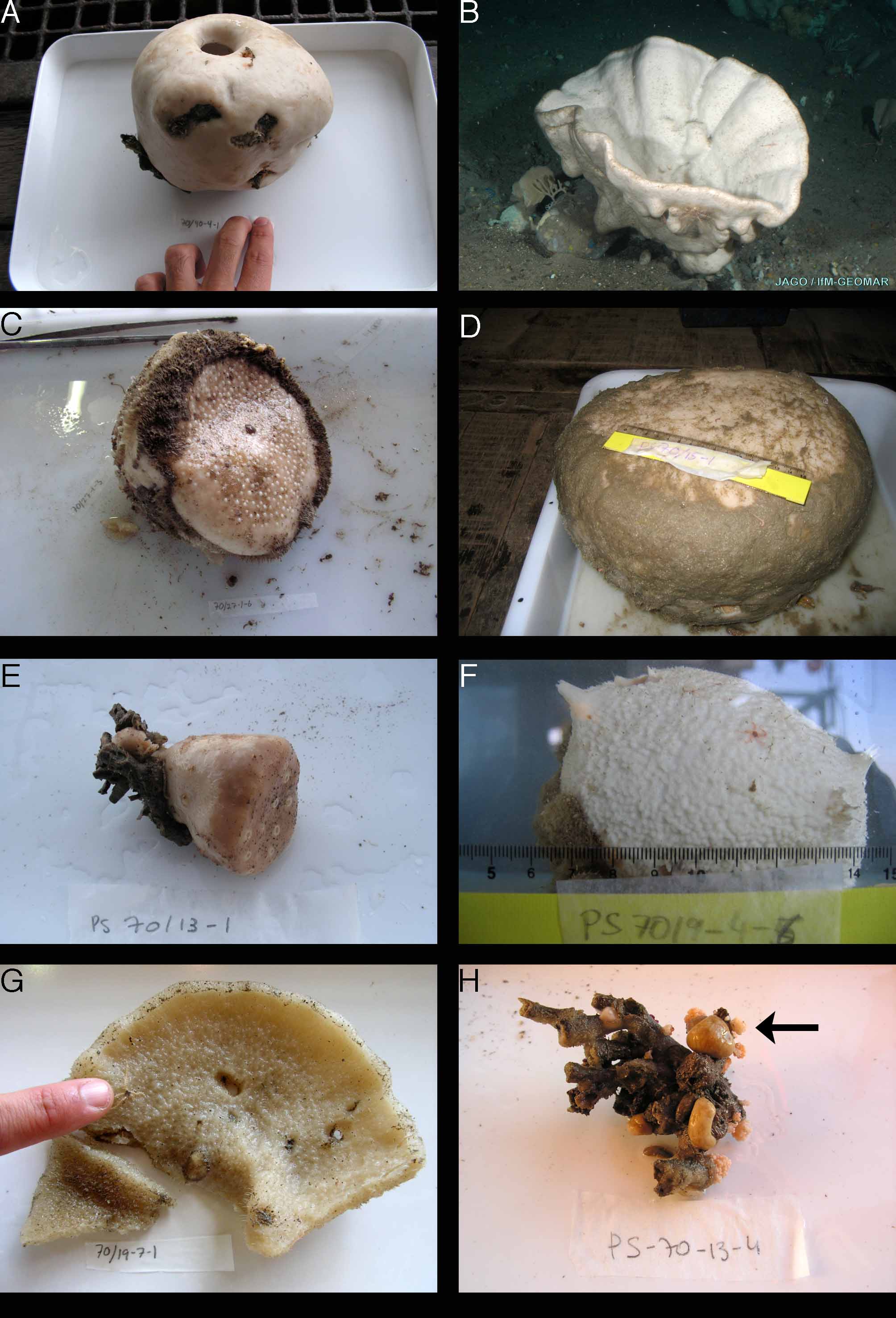


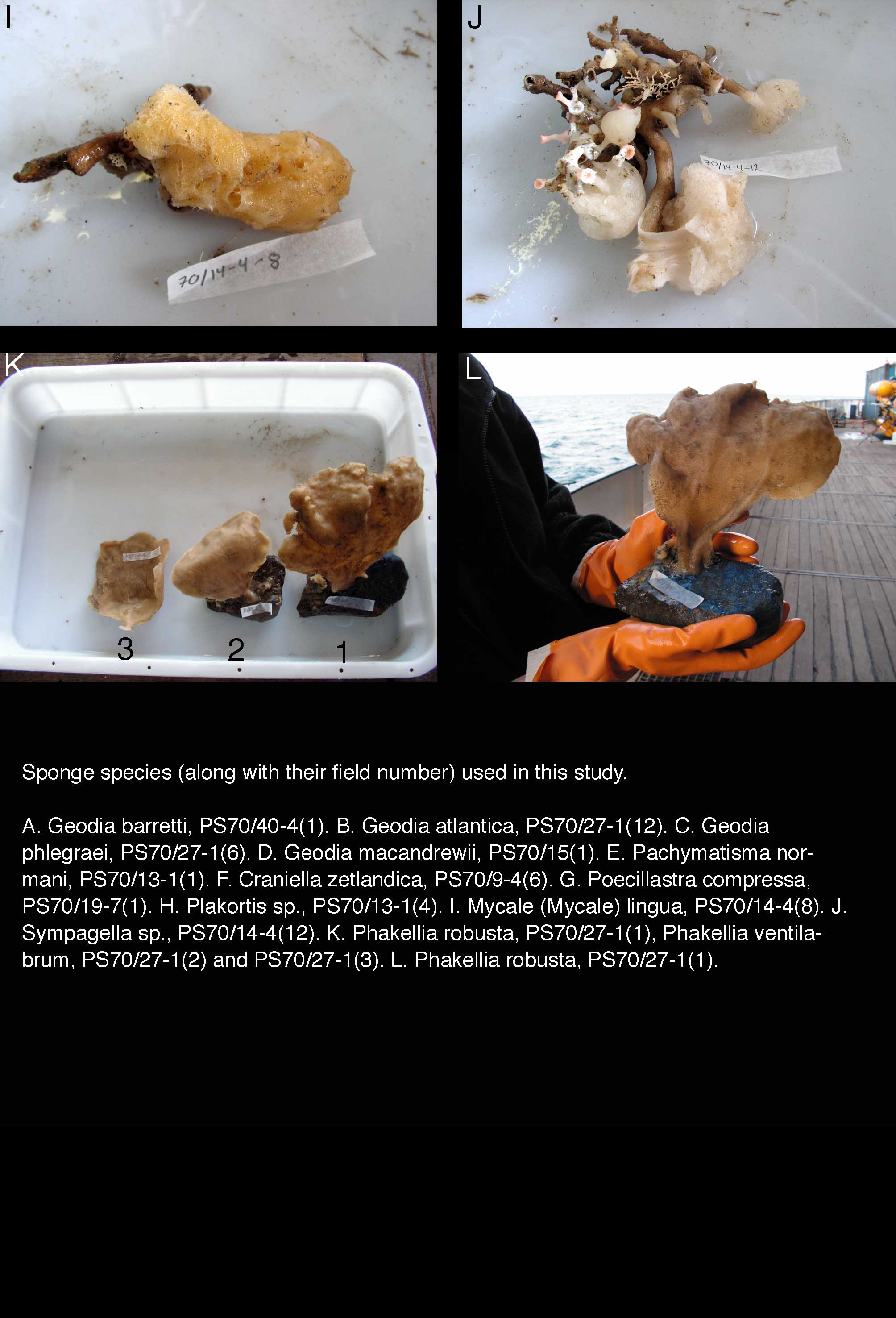


Sponge species (along with their field number) used in this study.

**A.** *Geodia barretti*, PS70/40-4(1). **B.** *Geodia atlantica*, PS70/27-1(12). **C.** *Geodia phlegraei*, PS70/27-1(6). **D.** *Geodia macandrewii*, PS70/15(1). **E.** *Pachymatisma normani*, PS70/13-1(1). **F.** *Craniella zetlandica*, PS70/9-4(6). **G.** *Poecillastra compressa*, PS70/19-7(1). **H.** *Plakortis* sp., PS70/13-1(4). **I.** *Mycale (Mycale) lingua*, PS70/14-4(8). **J.** *Sympagella* sp., PS70/14-4(12). **K.** *Phakellia robusta*, PS70/27-1(1), *Phakellia ventilabrum*, PS70/27-1(2) and PS70/27-1(3). **L.** *Phakellia robusta*, PS70/27-1(1).
